# Effects of individualized electrical impedance tomography and image reconstruction settings upon the assessment of regional ventilation distribution: Comparison to 4-dimensional computed tomography in a porcine model

**DOI:** 10.1371/journal.pone.0182215

**Published:** 2017-08-01

**Authors:** Florian Thürk, Stefan Boehme, Daniel Mudrak, Stefan Kampusch, Alice Wielandner, Helmut Prosch, Christina Braun, Frédéric P. R. Toemboel, Johannes Hofmanninger, Eugenijus Kaniusas

**Affiliations:** 1 Institute of Electrodynamics, Microwave and Circuit Engineering, TU Wien, Vienna, Austria; 2 Department of Anesthesia, Pain Management and General Intensive Care Medicine, Medical University of Vienna, Vienna, Austria; 3 Department of Biomedical Imaging and Image Guided Therapy, Medical University of Vienna, Vienna, Austria; 4 Anesthesiology & Perioperative Intensive Care Medicine, University of Veterinary Medicine Vienna, Vienna, Austria; University of Houston, UNITED STATES

## Abstract

Electrical impedance tomography (EIT) is a promising imaging technique for bedside monitoring of lung function. It is easily applicable, cheap and requires no ionizing radiation, but clinical interpretation of EIT-images is still not standardized. One of the reasons for this is the ill-posed nature of EIT, allowing a range of possible images to be produced–rather than a single explicit solution. Thus, to further advance the EIT technology for clinical application, thorough examinations of EIT-image reconstruction settings–i.e., mathematical parameters and addition of a priori (e.g., anatomical) information–is essential. In the present work, regional ventilation distribution profiles derived from different EIT finite-element reconstruction models and settings (for GREIT and Gauss Newton) were compared to regional aeration profiles assessed by the gold-standard of 4-dimensional computed tomography (4DCT) by calculating the root mean squared error (*RMSE*). Specifically, non-individualized reconstruction models (based on circular and averaged thoracic contours) and individualized reconstruction models (based on true thoracic contours) were compared. Our results suggest that GREIT with noise figure of 0.15 and non-uniform background works best for the assessment of regional ventilation distribution by EIT, as verified versus 4DCT. Furthermore, the *RMSE* of anteroposterior ventilation profiles decreased from 2.53±0.62% to 1.67±0.49% while correlation increased from 0.77 to 0.89 after embedding anatomical information into the reconstruction models. In conclusion, the present work reveals that anatomically enhanced EIT-image reconstruction is superior to non-individualized reconstruction models, but further investigations in humans, so as to standardize reconstruction settings, is warranted.

## Introduction

Assessing lung function in patients with failing lungs is essential for personalized optimization of ventilator settings–and subsequently, for their clinical outcome [1]. Commonly, global parameters, such as the compliance of the respiratory system, are directly assessed at the patient’s bedside [2,3]. More detailed information on the regional lung condition, on the other hand, is provided by modern imaging technologies. Computed tomography (CT) and other sophisticated radiologic imaging techniques make highly-resolved spatial information accessible, but they yield only single point measurements, require relocation of the patient and are associated with radiation exposure.

A promising technology for the dynamic bedside monitoring of lung function is electrical impedance tomography (EIT), a non-invasive and radiation-free imaging modality. EIT provides 2-dimensional images at high temporal resolution (up to 50 Hz), describing thoracic impedance changes mainly caused by tidal ventilation [4]. The working principle is based on repetitive, rotating injection and measurement of small currents and voltages, respectively, from surface electrodes attached around the thorax. Sophisticated reconstruction algorithms are then applied to obtain EIT-images of low spatial resolution (usually 32x32 pixels) [5].

Various studies have validated the ability of EIT to display regional ventilation distribution against established imaging modalities like dynamic CT [6], positron-emission tomography [7], single photon emission CT [8], or XENON CT [9]. Furthermore, parameters such as regional lung compliance [10], regional over-distention [11], lung inhomogeneity [12] or cyclic recruitment [13] can be accurately determined by EIT images, but require defined ventilation maneuvers (e.g., slow inflation, or defined pressure trials).

Despite the above-mentioned potential to dynamically monitor lung function, EIT has not yet been adopted in the routine clinical setting. This is due to the fact that correct interpretation of basic EIT-images requires expert knowledge, and that large clinical trials demonstrating the benefit of EIT-guided ventilation are still missing. Moreover, easily comprehensible and clinically meaningful parameters–produced without the need for specific ventilation maneuvers–are desperately needed. Among several technical challenges of EIT that need to be addressed in order to achieve this goal, one critical issue remains the ill-posed nature of the EIT-image reconstruction. As electrical currents branch out inside the body [14], no unique solution for the inverse problem exists [15] and the generated EIT-images highly depend upon reconstruction algorithms and their settings [16]. Usually a finite element model (FEM) is utilized to compute an approximate (forward) solution for the given spatial domain prior to the reconstruction process [17]. Inaccurate shapes of this FEM highly influence the resulting EIT-image, due to mismatching electrical field simulations [18].

In order to enhance the information content and improve the positional accuracy of EIT-images, it has been proposed to embed a priori anatomical information–such as thoracic body contours and lung boundaries–into EIT-image reconstruction [18,19]. Although some efforts have been made to provide averaged contour models in regard to anthropometric data for different species [20], most commercially available clinical devices still rely on inaccurate models [18], and thorough clinical validation studies are missing. Additionally, since no standard has yet been established in EIT reconstruction, different reconstruction algorithms and settings further increase the number of possible EIT-images [16,21]. Recently, the EIT community tried to approach this problem by defining certain quantifiable figures of merit within the Graz consensus Reconstruction algorithm for EIT (GREIT) [22]. Even so, various reconstruction parameters (e.g., regularization term) can still be selected arbitrarily, whereby their influence on the resulting EIT-images and derived parameters has not yet been comprehensively analyzed in-vivo.

In order to address the issues of EIT-image reconstruction, the aim of the present study was first to assess the impact of reconstruction settings for GREIT and Gauss Newton, and to identify optimal reconstruction settings. Secondly, applying these settings, we investigated the impact of different reconstruction models in their ability to assess regional ventilation distribution by comparing the results to the gold-standard technique of 4-dimensional CT (4DCT). All measurements were performed in a piglet animal model because of the high anatomical similarity to human lungs. Tidal ventilation EIT-images were reconstructed by (1) standard circular shape, (2) averaged thoracic body contours, and (3) individualized thoracic body contours. Moreover, EIT-images derived from (2) and (3) were further enhanced by integration of anatomical lung borders to extract pulmonary EIT-pixels only.

## Material and methods

After approval by the animal ethics committee of the Medical University of Vienna (ethics approval No. 53/11), N = 13 landrace piglets (average age 81±22 days; weight: 29±6 kg) in total were investigated. Two animals were necessary to implement the setup and the study protocol. A further three animals were required to investigate the impact of the different EIT-image reconstruction settings. Model comparisons were conducted in eight animals. Thus, N = 11 animals could be included for analysis. The present study was carried out in strict accordance with the recommendations in the Guide for the Care and Use of Laboratory Animals of the National Institutes of Health, and all efforts were made to avoid animal suffering [23]. All animal experiments were performed at the facilities of the University of Veterinary Medicine, Vienna, Austria.

### Experimental setup and animal treatment

On the day of the experiments, the animals were sedated at the animal housing facilities via intramuscular injection of 0.4 mg kg^-1^ atropine, 8 mg kg^-1^ azaperone, 0.2 mg kg^-1^ midazolam and 3 mg kg^-1^ s-ketamine. Thereafter, the animals were transported to the operation room, where general anesthesia was induced and maintained with intravenous 10–20 mg kg^-1^ hr^-1^ propofol and 5–10 μg kg^-1^ hr^-1^ fentanyl. After orotracheal intubation (7.5 to 8.5 ID) the animals were mechanically ventilated (Elisa 800, SALVIA medical GmbH, Germany) in pressure-controlled mode using a protective ventilation regime: Plateau pressure (P_Insp_) was set so as to achieve a tidal volume (V_T_) of 6 ml/kg body weight; a positive end-expiratory pressure (PEEP) was set to a level of 5 cm H_2_O; the fraction of inspired oxygen was set to 0.4; the respiratory rate was adjusted to maintain normocapnia, resulting in 20–30 breaths per minute. Arterial and central venous lines were placed using ultrasound-guided puncture and the Seldinger technique to gain vascular access for routine monitoring. The animals’ body temperatures were kept between 38°C and 39°C via body surface warming. Animals were monitored continuously for depth of anesthesia and cardiopulmonary stability.

Thereafter, the animals were transferred to the CT facility (Emotion 16, Siemens, Germany) and routine monitoring of respiration and hemodynamic parameters was implemented using an Infinity® Delta Monitor (Infinity® Delta Monitor, Dräger Medical, Lübeck, Germany). Dynamic multi-detector (16-slice) CT (4DCT) measurements were performed with slice collimation of 16x0.6 mm and a gantry rotation time of 0.6 seconds, resulting in volume stacks of 4.8 mm in height at a sampling frequency of 1 Hz. The tube potential was set to 70–80 kV and no contrast agent was applied.

The EIT sensor belt was attached around the thorax at approximately the 6^th^ to 8^th^ intercostal space and connected to the EIT measurement device (EIT Pioneer Set, Swisstom AG, Landquart, Switzerland). For this study, we used custom-built EIT sensor belts with 32 active electrodes, which were manufactured on request by Swisstom (Swisstom AG, Landquart, Switzerland). What set the custom-built EIT sensor belt apart from the commercially distributed ones was the connection to active amplification was placed approximately 15 cm below the surface electrode plane. This was achieved by enlarging the conductance textile material of the sensor belt and by isolation of the respective electrodes, so as to avoid any noise during CT acquisition from the metallic components of the amplification circuit. Thus, this custom-built EIT belt had no influence on radiation attenuation and was well suited for synchronized EIT and 4DCT measurements. EIT Measurements were performed with 3 mA injection current at 195 kHz, skip 4 measurement technique [24] and a 48 Hz sampling frequency.

Before performing the measurements, all recording devices were synchronized to the time-stamp of the CT scanner. At the end of the experiments, the animals were euthanized under deep anesthesia by an overdose of propofol, fentanyl, and potassium chloride.

### Study protocol

Prior to the measurements, an initial CT topogram and a baseline volume CT scan were performed. Then, the CT table position was set to match the central EIT sensor belt position and was fixed for 4DCT measurements. Five minutes prior to the synchronized measurements of EIT and 4DCT, mechanical ventilation was set as follows: P_insp_ was adjusted to obtain a V_T_ of 10 ml/kg body weight; respiration rate was set to 6 min^-1^; inspiration-to-expiration time was set to 1:1; a PEEP of 5 cm H_2_O was dialed in. Then, EIT and 4DCT recordings were captured synchronously over the course of three consecutive breathing cycles at a sampling frequency of 48 Hz and 1 Hz, respectively. This resulted in a measurement duration of at least 30 seconds, with approximately 30 CT lung stacks and 1,440 EIT frames captured over the recording time period. The time courses (in Hounsfield Units ***HU***(*t*) by 4DCT and in voltage ***u***(*t*) by EIT) were digitally stored for further post-processing. After the 4DCT measurement, an additional volume CT scan was performed during an inspiratory breath hold from the level of the cervicothoracic transition (i.e., above the lung apex) to the liver. This scan was later used to extract the anatomical information for modeling the individualized FEM.

### Model generation and EIT-image reconstruction

**Fig 1:**
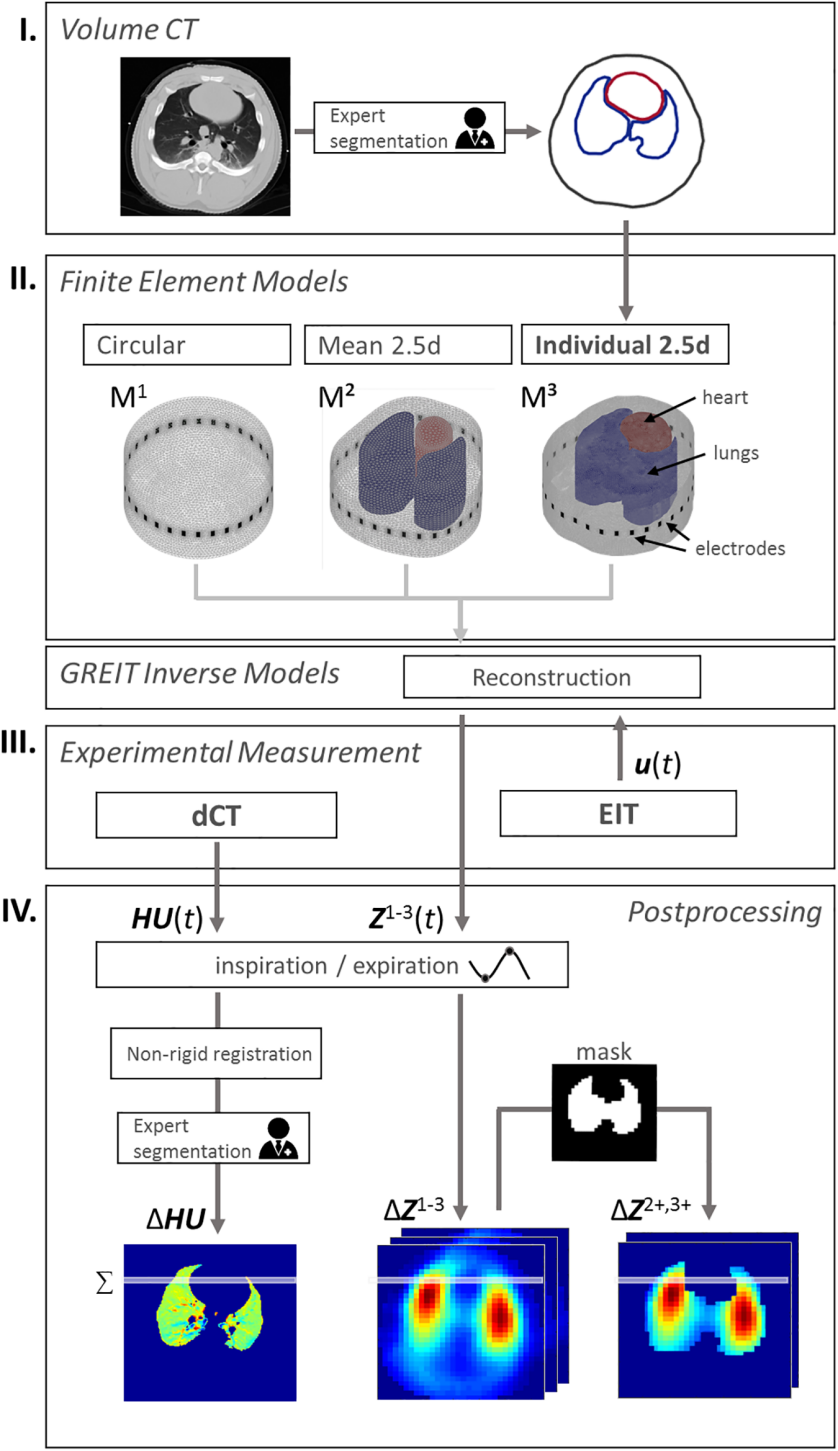
Acquisition and processing procedure. I) Volume CTs were recorded for each pig and the contours of thorax, lungs and heart were extracted by radiologists. II) Finite element models were created using no prior information (M^1^), averaged contours (M^2^) or individual contours of each animal (M^3^). These models and optimized reconstruction settings were utilized to calculate the reconstruction matrix of EIT. III) Experimental measurement of 4DCT and EIT as well as reconstruction of EIT image series ***Z***^1-3^(*t*) and (IV) extraction of tidal volume images and their ventilation profiles.

Images were reconstructed based on three different types of FEMs. A circular FEM (M^1^) without any anatomical information and an averaged FEM for pigs (as previously described in [20]) (M^2^) were utilized as non-individualized models. Additionally, an individualized FEM (M^3^) was created for each pig based on anatomical information (compare Fig 1II). For this purpose, the volume CT scans during the inspiratory hold were used to extract the individual contours of thorax, lungs and heart (see Fig 1I) at the EIT sensor belt level. This segmentation procedure was performed manually for each pig and controlled by a second radiologists. The mismatch of model contours between M^1^, M^2^ and M^3^ was quantified using the symmetric difference [18]. For this purpose, the thorax contours were aligned based on their gravitational center and normalized to the same area (*π*). The relative error Δ*S* was then calculated as the ratio of non-overlapping area to *π* (see S1 Fig).

In order to better match the nature of three-dimensional current density distribution through the thorax, FEMs were extruded to obtain 2.5D models [25] with approximately 200k elements. Finally, GREIT [22] inverse models were created for each FEM using optimized parameters (from the results section Model Comparison), yielding a total of 8 individual anatomically-enhanced models (one for each pig) and 2 general reconstruction models. Lungs and heart in M^2^ and M^3^ were weighted with 0.2 and 1.5, respectively, to account for their different conductivities, as described in [26]. For each voltage measurement ***u***(*t*), images were reconstructed using the described models to obtain impedance distributions ***Z***^1-3^(*t*) ∈ ℝ^32x32^, as related to the respective models M^1-3^. All calculations were performed using a combination of proprietary MATLAB 2016a (The MathWorks Inc., Natick, MA, USA) scripts, the open source frameworks EIDORS 3.71 [27] and NETGEN [28].

### Evaluation of reconstruction settings

**Fig 2:**
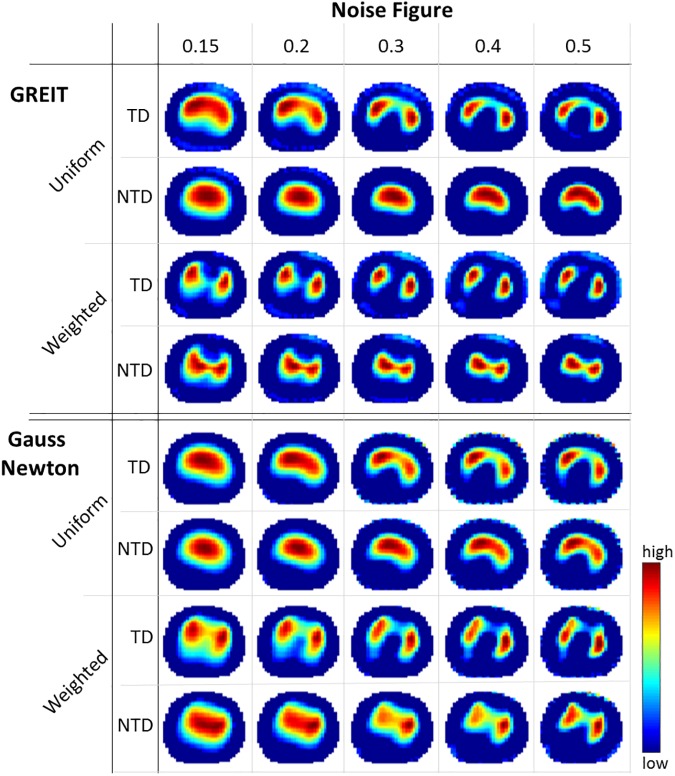
Reconstructed EIT images. Reconstructed EIT images showing tidal impedance changes (red = high, blue = low) using different settings for GREIT and Gauss Newton. Noise figure controls smoothing of the images, time difference (TD) and normalized time difference (NTD) are voltage reference methods, and weighted reconstruction–in contrast to uniform reconstruction–considers that lungs are less conductive than the surrounding tissue. Images are zeroed at a threshold of 10% to reduce artifacts.

Similar to previous work [29], a systematic investigation of the reconstruction parameters was performed, in order to optimize reconstruction settings. Specifically, GREIT and Gauss Newton (GN) algorithms with different settings were compared in three pigs using their anatomically-enhanced FEM, M^3^. For both algorithms, noise figure *nf*, background uniformity (uniform or weighted lungs and heart [26]) and voltage reference method were varied. Difference voltage can be either calculated as time difference (TD) *v*_diff_ = *v* − *v*(*t*_r_), or normalized time difference (NTD) $v_{diff}=\frac{v}{v(t_{r})}-1$, with *t*_r_ as an arbitrary reference time instant (one reference per measurement) [26]. Note that for GN, the hyperparameter–determining the level of regularization–was automatically chosen to match the given *nf* [30]. For GREIT, two additional parameters—the weighting radius *rw* and the target size *ts* of test samples—were considered (see Table 1 for all parameters). From each of the resulting 3010 parameter variations of M^3^, images were reconstructed for three pigs (a selection can be seen in Fig 2) and the correlation between tidal images of CT and EIT (see section Post-Processing of EIT and CT for more details) was calculated. The identified reconstruction parameters were then applied to all models M^1-3^ as described earlier.

**Table 1:** Reconstruction settings.

	Noise Figure	Background	Reference	Target Size	Weighting Radius	Prior
**GREIT**	0.1–0.5	uniform / weighted	TD / NTD	0.1–0.9	0.1–0.5	
**Gauss Newton**						Laplace / Tikhonov / Noser

Reconstruction parameters for GREIT and Gauss Newton. TD—time difference, NTD—normalized time difference

### Post-processing of EIT and CT

**Fig 3:**
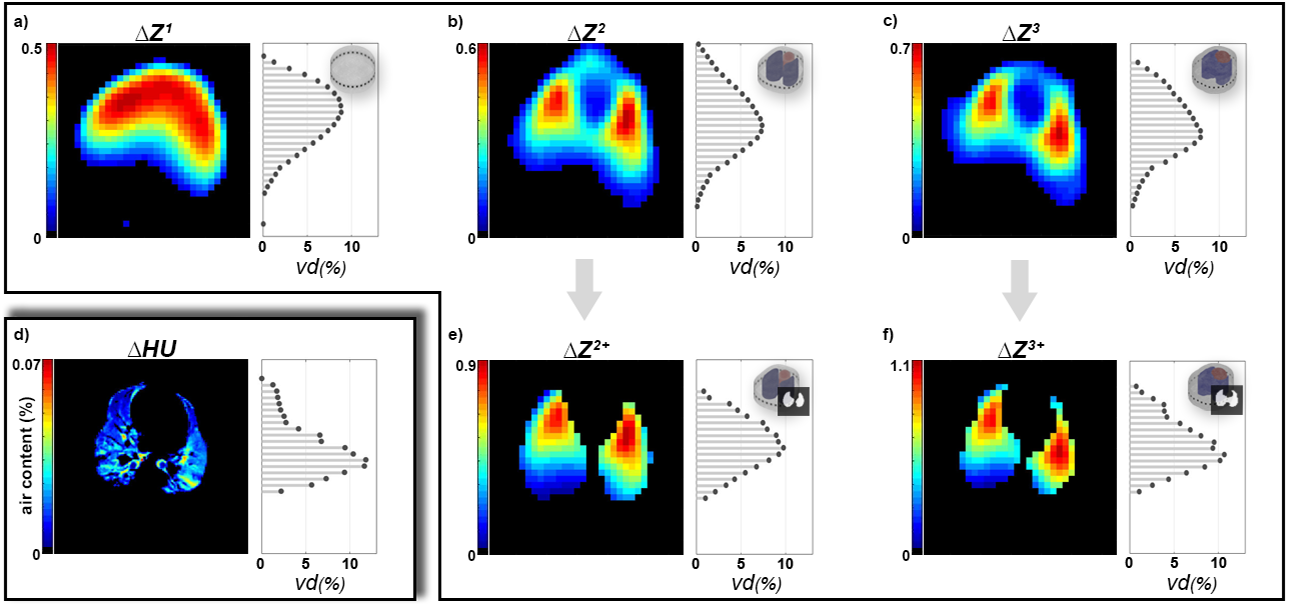
Tidal volume images. Tidal volume images and anteroposterior ventilation distribution for (a-c) EIT from M^1^-M^3^, (e) EIT with averaged reconstruction model M^2^ and lung mask, (f) EIT with individual reconstruction model M^3^ and lung mask, and (d) the reference 4DCT. Note that relative values for Δ***HU*** are lower due to the higher spatial resolution.

End-inspiration and end-expiration time instants, *t*_in_ and *t*_ex_, were detected from averaged ***Z***(*t*) and ***HU***(*t*) signals. Since the reconstruction model in EIT is static and works as a spatial reference space to which the relative impedance changes are mapped, tidal images can be defined as Δ***Z*** = ***Z***(*t*_in_)—***Z***(*t*_ex_). A robust tidal image was obtained by averaging all Δ***Z*** during CT measurement. For tidal images Δ***Z***^2,3^, only pulmonary pixels (from lung contours derived from CT) were selected to obtain Δ***Z***^2+^ and Δ***Z***^3+^ (see Fig 3E and 3F). If no anatomical information was used for post-processing, pulmonary pixels were determined by excluding pixels below a threshold of 10% of the maximum of Δ***Z*** to reduce non-ventilation artifacts.

In contrast to EIT, 4DCT images are not spatially normalized and show complex non-linear movements of the lungs, chest and heart. In order to calculate comparable tidal ventilation distributions based on 4DCT, a spatial mapping of the end-inspiratory image ***HU***(*t*_in_) to the end-expiratory image ***HU***(*t*_ex_) is required. We utilized a non-rigid image registration algorithm [31] with high regularization to perform this mapping. Specifically, a non-linear transformation **T** was performed so that ***HU***(*t*_in_) ≈ **T**(***HU***(*t*_ex_)) and the tidal image Δ***HU*** could be calculated by Δ***HU = HU***(*t*_in_)—**T**(***HU***(*t*_ex_)). To eliminate artifacts from cardiac movement and CT reconstruction errors, all images at *t*_in_ and *t*_ex_, respectively, were averaged prior to registration. Furthermore, anatomical lung regions in Δ***HU*** were identified manually by a radiologist as to decrease the influence of possible registration errors outside of the lungs.

### Regional tidal ventilation profiles by 4DCT and EIT

To directly compare the ventilation distributions of 4DCT and EIT, 32 horizontal regions were defined for both Δ***HU*** and Δ***Z–***thus matching the native resolution of the EIT-images (see Fig 1IV). The pixel values of each region were then summed up and normalized to calculate the anteroposterior ventilation distributions *vd*^CT^ and *vd*^EIT^_,_ whereby the latter represents *vd*^1^, *vd*^2^, *vd*^3^, *vd*^2+^ and *vd*^3+^.

### Statistics

To compare the ventilation distribution profiles assessed by the different EIT-image reconstructions versus those derived from 4DCT, the root mean square errors, *RMSE*, between *vd*^CT^ and *vd*^EIT^ were calculated for each pig. For statistical assessment of the differences in *RMSE* between EIT and 4DCT, a Kruskal–Wallis test and Tukey-Kramer’s procedure for multiple comparisons were used. Pearson correlations and Bland-Altman analysis were performed as descriptive statistics to assess the similarity and agreement between the EIT results and the gold standard of 4DCT.

## Results

### Comparison of reconstruction settings

As depicted in Fig 2, reconstruction settings strongly influenced the resulting tidal ventilation EIT-images Δ***Z***. Without weighting (uniform background) of lung regions, in both GREIT and GN, the center of ventilation moved to the anterior site (compare TD weighted and uniform at *nf* = 0.15 for GREIT in Fig 2), while the right and left lung could not be clearly distinguished (except for *nf* above 0.3 and TD). Impedance changes were pulled towards the center of the image for NTD, producing reasonable images only for non-uniform background. For GREIT, decreasing *ts* with non-uniform background slightly increased the area of impedance changes in the reconstructed images, particularly in the anteroposterior axis (not shown). This effect could not be seen with uniform background and was also less pronounced for NTD. Similar blurring occurred with increasing *rw*, but with stronger lateral expression. Permutations with *rw* values below 0.15 and above 0.3 produced strong artifacts and severe distortion of the images (see S2 Fig). Differences between GN and GR were most visible for non-uniform background with a shift of ventilation activity towards the anterior site in GN. In addition, all reconstructions in GN showed stronger artifacts at electrode positions. Our results suggest the use of the GREIT algorithm with weighted lungs and heart, *nf* of 0.15, *ts* of 0.06, *rw* of 0.15 and TD as reference method. The corresponding maximum 2D correlation with Δ***HU*** images for this combination and M^3^ was 0.69 (compare Table 2). For further verification of this first independent analysis, 4 additional pigs from the model comparison group were investigated post-hoc. While here, the top reconstruction setting turned out to be slightly different (GREIT with *nf* of 0.15, *ts* of 0.08, *rw* of 0.15 with NTD), our initial parameter setting was still rank 10 with a high correlation coefficient (0.66 vs. 0.681). See S1 and S2 Tables for top rankings of reconstruction algorithms.

**Table 2:** Correlation of different reconstruction settings.

			Noise Figure, *nf*				
			*0*.*15*	*0*.*2*	*0*.*3*	*0*.*4*	*0*.*5*
GREIT	Uniform	TD	0.69	0.63	0.57	0.55	0.54
		NTD	0.67	0.55	0.47	0.44	0.43
	Weighted	TD	0.50	0.50	0.54	0.56	0.57
		NTD	0.46	0.41	0.36	0.36	0.37
Gauss Newton	Uniform	TD	0.52	0.52	0.52	0.49	0.45
		NTD	0.48	0.47	0.45	0.43	0.39
	Weighted	TD	0.43	0.44	0.42	0.42	0.43
		NTD	0.43	0.42	0.39	0.39	0.40

Pearson correlation coefficients between tidal images from 4DCT and EIT images originating from different reconstruction settings for M^3^. The highest similarity was identified with GREIT at *nf* = 0.15, *ts* = 0.06, *rw* = 0.15, TD with weighted lungs and heart. Note that for this representation *ts* and *rw* as well as the prior (Laplace) for Gauss Newton were fixed.

### Model comparison

**Fig 4:**
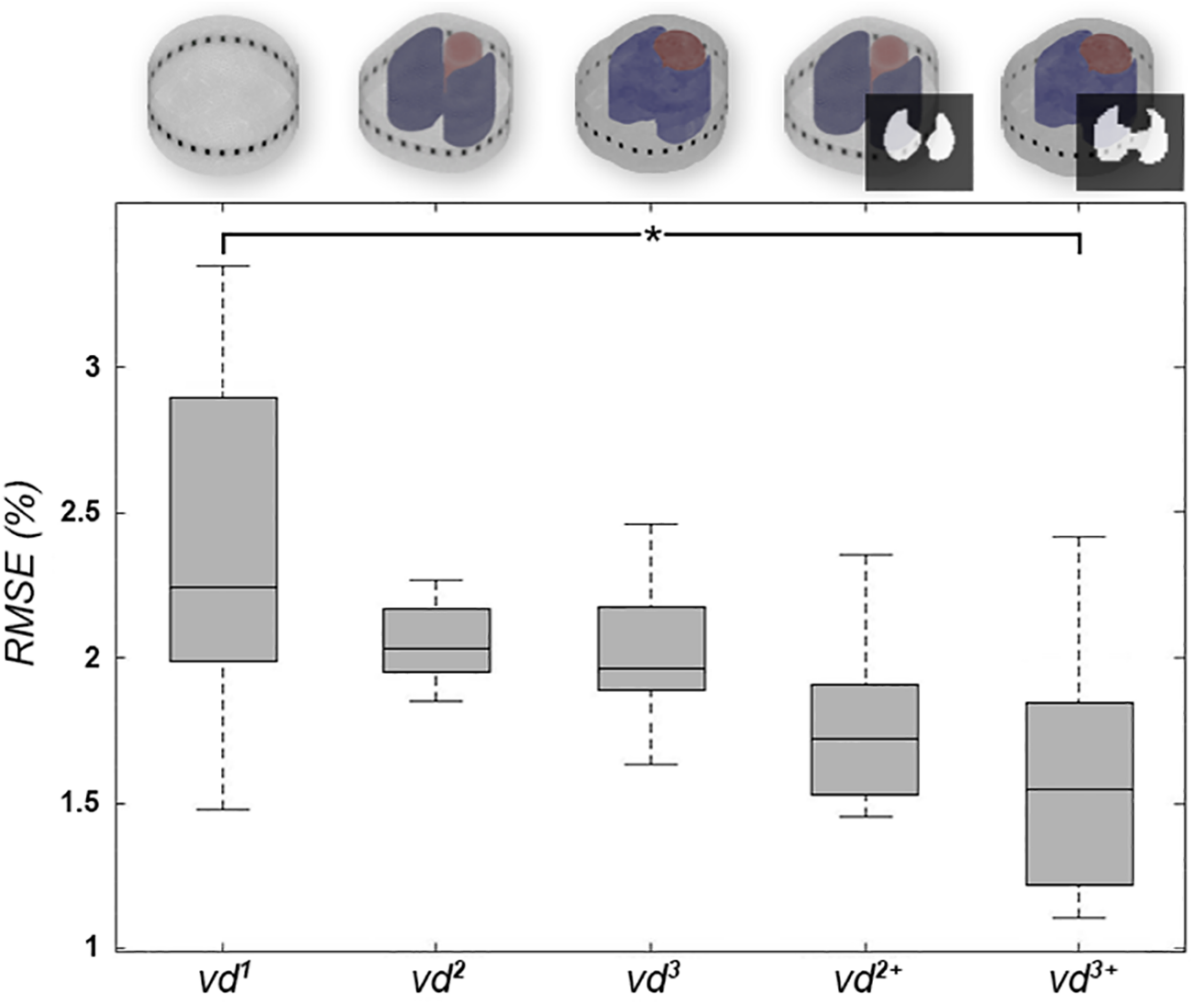
RMSE values of all animals. Boxplots for *RMSE* values over all pigs (n = 8). The circular model (*vd*^1^) showed high variation and high error, whereas *RMSE* decreased with the addition of anatomical information in *vd*^2^, *vd*^2+^ and *vd*^3^. *RMSE* was significantly lower after adding further individual anatomical information in *vd* ^3+^ (p < 0.03).

**Fig 5:**
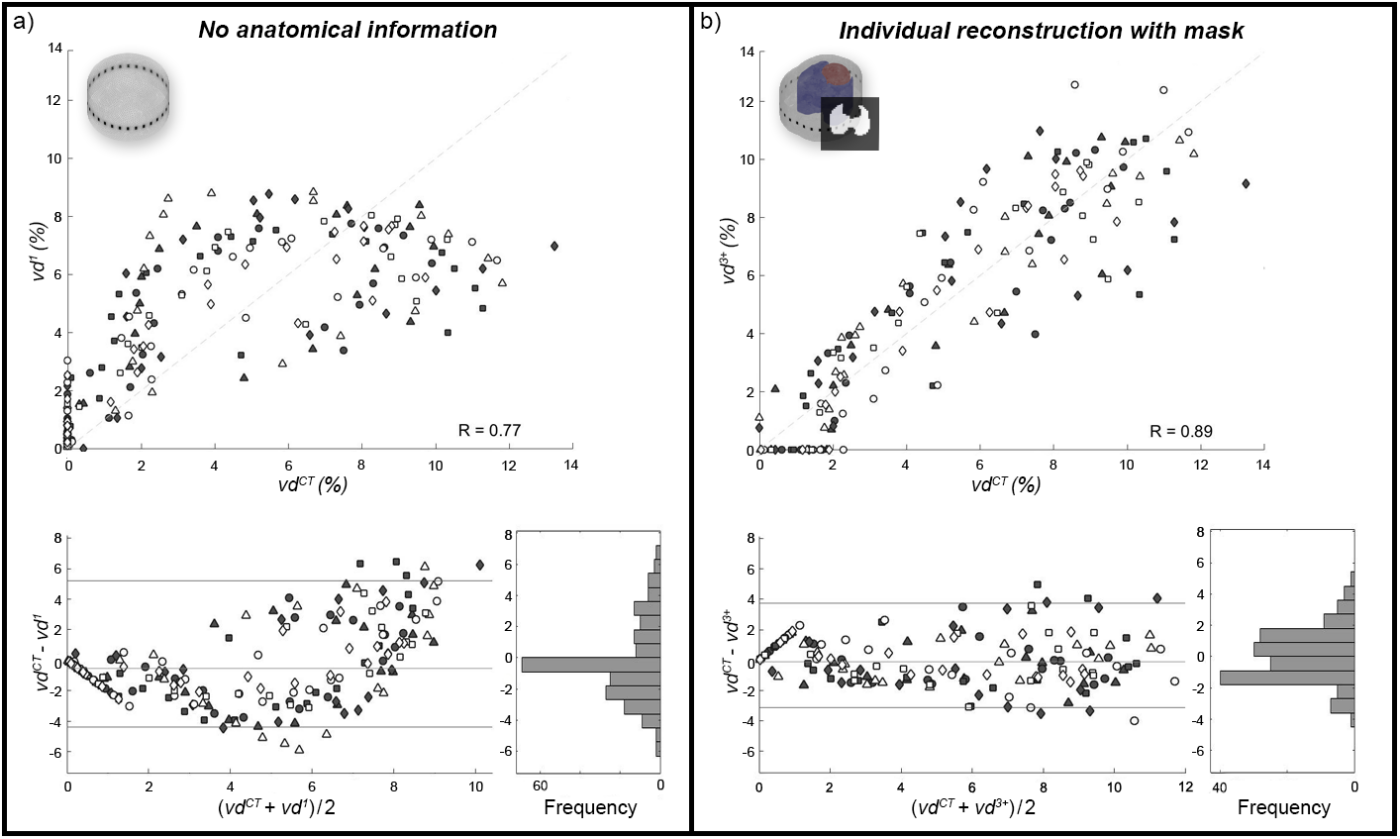
Regional ventilation in 4DCT and EIT. Comparison of regional ventilation acquired from 4DCT and EIT. a) Calculations based on the circular model M^1^ without anatomical information and (b) on M^3^ with individual boundaries and lung mask. For both, pooled Pearson correlation, Bland-Altman and distribution of differences are shown. Different symbols correspond to values from different animals (n = 8), whereby only values of *vd* greater than zero in at least one of the two compared methods are considered. Since the differences of both methods are not normally distributed, bias and limits of agreement are represented as median and 95% quantile interval, respectively.

The geometry mismatch, Δ*S*, between individual contours in M^3^ and non-individual contours in M^1^ and M^2^ were 7.66 ± 1.57% and 5.00 ± 0.96%, respectively (see S3 Table for more details). Between M^1^ and M^2^, Δ*S* was 8.61%. The anteroposterior ventilation distribution profiles results derived from the different EIT-reconstruction models were compared by *RSME* versus the results of the gold-standard method of 4DCT. Here, *RMSE* was highest in *vd*^1^ with 2.53±0.62%, decreased with increasing anatomical information and was significantly lower for *vd*^3+^ with 1.67±0.49% (p < 0.03). The detailed results appear in Fig 4 and Table 3. Considering descriptive statistical analysis, pooled Pearson correlation coefficients were 0.77, 0.88 and 0.89 for *vd*^1^, *vd*^2^ and *vd*^3^, respectively. While there was no difference from *vd*^3^ to *vd*^3+^, *vd* to *vd*^2+^ decreased slightly to 0.87. Fig 5 highlights the results for *vd*^1^ and *vd*^3+^. The Bland-Altman analysis revealed similar results of agreement, with bias (and 95% confidence interval of quantile) decreasing from -0.54% (9.58%) to -0.03% (6.86%), from *vd*^1^ to *vd*^3+^. For a data summary of distributions of *vd* based on the different models, see S3 Fig and S4 Table.

**Table 3:** Individual root mean square errors.

Animal	*vd*^1^	*vd* ^2^	*vd* ^3^	*vd* ^2+^	*vd* ^3+^
1	2.41	2.02	1.95	1.57	1.52
2	2.07	1.92	1.88	1.45	1.33
3	2.85	2.82	2.46	2.36	1.97
4	3.22	2.84	2.74	2.71	2.34
5	2.94	1.99	2.33	1.82	2.42
6	3.34	2.26	1.97	1.94	1.10
7	1.91	2.07	2.02	1.62	1.57
8	1.48	1.85	1.63	1.49	1.10
	2.53 ± 0.62	2.22 ± 0.37	2.12 ± 0.33	1.87 ± 0.42	1.67 ± 0.49

Individual root mean square errors (in %) for different profiles generated from models with increasing anatomical information; *vd*^1^ … circular, *vd*^2^ … averaged, *vd*^3^ … individual, *vd*^2+^ … averaged with only pulmonary pixels, *vd*^3+^ individual with only pulmonary pixels.

## Discussion

In this work, the influence of different settings for EIT image reconstruction on the assessment of regional ventilation distribution was evaluated versus the gold standard technique of 4DCT. Specifically, reconstruction parameters for GREIT and Gauss Newton algorithms were evaluated; optimal settings were identified (GREIT with *nf* of 0.15, *ts* of 0.06, *rw* of 0.15 and weighted lung and heart regions) and applied to a circular, an averaged and a novel individualized reconstruction model. EIT-images reconstructed by averaged and individualized models were further enhanced with anatomical lung contours to identify pulmonary pixels. Tidal anteroposterior ventilation distribution profiles were calculated for all EIT-images and for the 4DCT scans. Direct comparison between the different models and 4DCT was carried out by calculation of *RMSE*. Our results showed that the error was highest for the circular model and lowest for the individual model—with 2.53±0.62% and 1.67±0.49%, respectively. Analogously, correlation between EIT and 4DCT was highest for the anatomically-enhanced image reconstruction method.

Measurements were carried out in an experimental animal model using piglets, allowing for high-resolution, dynamic CT scanning over a long period of 30 seconds, with high radiation dosages. The experimental setup was designed for time-synchronized EIT and 4DCT measurements during ongoing mechanical ventilation. Since the 4DCT sampling frequency was rather low (1 Hz), respiratory rate was restricted to 6 min^-1^ in order to capture enough CT volume stacks over the course of a single respiratory cycle for clear identification of end-inspiratory and end-expiratory phases.

Additionally, the technical features of the CT scanner used a limited longitudinal coverage (the imaged lung stack) of only 4.8 mm. Although performed on the same thoracic level as the EIT belt, the volume imaged by 4DCT is therefore not equal to that covered by EIT. This is because the current density distribution is not limited to the 2D axial slice at the central EIT sensor belt position, but also extends several centimeters (approximately 3 cm) cranial and caudal–forming a lens-shape. However, it can be assumed that the functional behavior of the lungs along the anteroposterior axis in the healthy state is mostly independent from the cranio-caudal height [32].

Considering that EIT in its current form has not yet been adopted in clinical routine, we are convinced that enhancing EIT-images (e.g., by adding anatomical information extracted from CT scans) has the potential to facilitate the interpretation of EIT-images, and might allow the computation of novel and clinically meaningful parameters (e.g., Silent Spaces [33]). Still, EIT-image reconstruction remains a challenge, due to the ill-posed nature of EIT and the lack of clinical standards. Previous studies have investigated the influence of reconstruction methods on raw EIT images, as well as on derived physiological parameters [16], but comprehensive evaluations do not exist. While technical works give detailed explanations on the used reconstruction algorithm, they often rely on simulated data or EIT image analysis only [34]. In-vivo studies, on the other hand, trying to validate EIT versus a gold standard modality, mostly use commercial EIT systems with their implemented image reconstruction methods and fixed settings [6–9].

In contrast, this work establishes an experimental comparison as well as a validation of reconstruction methods. Here, we have to acknowledge that the sole use of the anatomically-enhanced FEMs (M^3^) to identify the optimal reconstruction settings might have biased our results. While it seems reasonable that more accurate geometries produce less errors and better spatial mapping [19], a comprehensive analysis of the other models (M^1^ and M^2^) should be performed in future works. In fact, reconstruction errors at the domain boundaries were highest in M^1^ and decreased for more accurate shapes in M^2^ and M^3^ (compare S4 Fig). It should be noted that for both, M^1^ and M^2^, Δ*S* was higher than 4%, which was previously considered as a reasonable threshold for reconstruction quality [18]. This highlights the importance of individualized EIT reconstruction. Another factor that could have influenced the results is the extraction of anatomical information from a volume CT scan performed during inspiratory hold, instead of the 4DCT images. We did so to prove the basic concept that a single volume CT (which is frequently conducted in clinical setting for diagnostic purposes) can be used in clinics for anatomical-enhancement of the EIT method. Determining the lung regions from static volume CT during inspiratory hold did not capture the movement in thoracic shape, lung and heart regions caused by tidal ventilation. Here, a combination of the presented method with other evaluation methods to assess moving lung borders or functional ROI methods [35] might be a valuable extension to our analysis. Other potential sources of errors are the choice of tissue property weighting (e.g., for lungs and heart) [36] and the exclusion of bones and other tissue properies in the FEMs, but a complete evaluation was beyond the scope of this work. Finally, while anesthetic drugs were equally dosed for each animal, the actual influence of these agents on hemodynamic mechanisms (e.g. pulmonary shunt or cardiac output) was not controlled.

Besides the results showing that anatomically-enhanced EIT was superior in mapping regional ventilation distribution compared to the more classical approaches, the embedded anatomical information also offers the opportunity to post-process novel and meaningful EIT-parameters in the future. Despite these promising results, further systematic investigations are needed in regard to different ventilation settings, different pathophysiologic lung conditions or even algorithms which were not taken into account by the present study (e.g., D-bar [37]), before our findings can be translated into clinical practice. Nonetheless, our novel introduced individualized EIT reconstruction model could be easily transferred to clinical routine–and applied in patients where a routine volume CT has been performed for other reasons (e.g. diagnostic purpose).

We conclude that appropriate reconstruction settings are crucial for the extraction of clinically relevant information, and that individualized (anatomically-enhanced) EIT image reconstruction offers considerable improvement over recently used, non-individualized reconstruction methods.

## Supporting information

S1 Fig Symmetric error of model geometries. Error of geometries for **(**a) individual (animal P03) versus circular model (Δ*S* = 8.47%), (b) individual versus mean model (Δ*S* = 4.33) and (c) contours of all individual models.
(TIF)

S2 Fig Undesirable EIT reconstructions. A collection of unphysiological EIT-images for certain reconstruction settings. Especially combinations containing *rw* below 0.1 and above 0.3 often generated distorted images from our data.
(TIF)

S3 Fig Visual data summary of anteroposterior profiles for model comparison. Data distribution of anteroposterior profiles for CT and EIT. Boxplots are given as median and 25^th^ and 75^th^ percentiles, respectively.
(TIF)

S4 Fig Artefacts at the boundary of different model geometries. a) Tidal volume images (animal P06) for circular (Δ*Z*1), averaged (Δ*Z*2) and individualized (Δ*Z*3) reconstruction model. b) After truncating the image above 10% of the maximum value, noise levels at the boundary become visible. The noise images are rectified for better visualization of noise levels (the color bar is only valid for (b)).
(TIF)

S5 Fig RMSE values of all animals for reconstruction settings based on previous results. Boxplots for RMSE values over all pigs (n = 8) using GREIT with *nf* = 0.15, *ts* = 0.05, *rw* = 0.25, TD and weighted lungs and heart. The circular model (*vd*^1^) showed high variation and high error, whereas RMSE decreased with the addition of anatomical information in *vd*^2^, *vd*^2+^ and *vd*^3^. RMSE was significantly lower after adding further individual anatomical information in *vd*^3+^ (p < 0.04).
(TIF)

S1 Table Top 15 rankings of reconstruction settings. Different reconstruction settings ranked by their 2d correlation values with 4DCT. Averaging the ranks of these settings provides a robust candidate for further analysis.
(DOCX)

S2 Table Validation of top rankings of reconstruction settings. Based on 4 animals of the model comparison group, a validation of the “optimal” reconstruction settings was performed in accordance with the previous evaluation. The previously identified settings appear within the top 10.
(DOCX)

S3 Table Geometry error of models. Individual error of model geometries for circular M^1^, mean M^2^ and individual M^3^ models. The error is defined as symmetric difference Δ*S*; i.e. non-overlapping regions of thorax contours divided by total area. As expected, Δ*S* was higher between M^1^ and M^3^ than between M^2^ and M^3^. All values are given in %.
(DOCX)

S4 Table Data summary of anteroposterior profiles for model comparison. Summary of average and standard deviation of anteroposterior ventilation distribution calculated from tidal volume images acquired by 4DCT and different EIT Models. All values are given in % as the fraction of horizontal region of interest (roi) ventilation to total ventilation. Approximate center of ventilations are written in bold letters.
(DOCX)

S1 File Data and scripts. (RAR)
