# Supplementary figures and images for: Effects of individualized electrical impedance tomography and image reconstruction settings upon the assessment of regional ventilation distribution: Comparison to 4-dimensional computed tomography in a porcine model
Source: PLoS One. 2017 Aug 1;12(8):e0182215. doi: 10.1371/journal.pone.0182215 (PMC5538699; doi:10.1371/journal.pone.0182215)

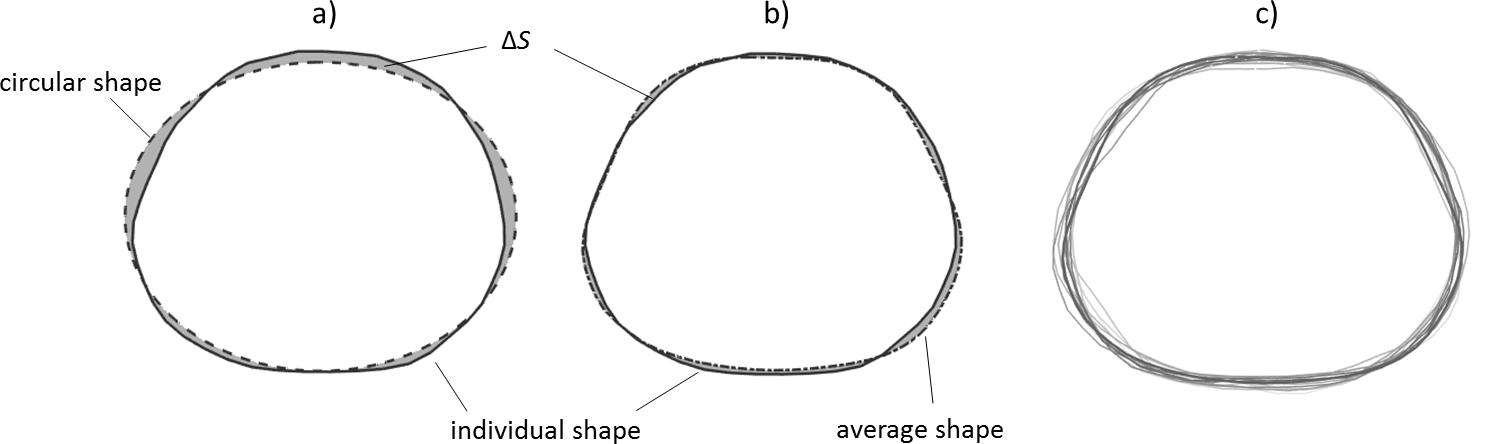

Supplement: S1 Fig — Error of geometries for (a) individual (animal P03) versus circular model (ΔS = 8.47%), (b) individual versus mean model (ΔS = 4.33) and (c) contours of all individual models. (TIF) [file pone.0182215.s001.tif]

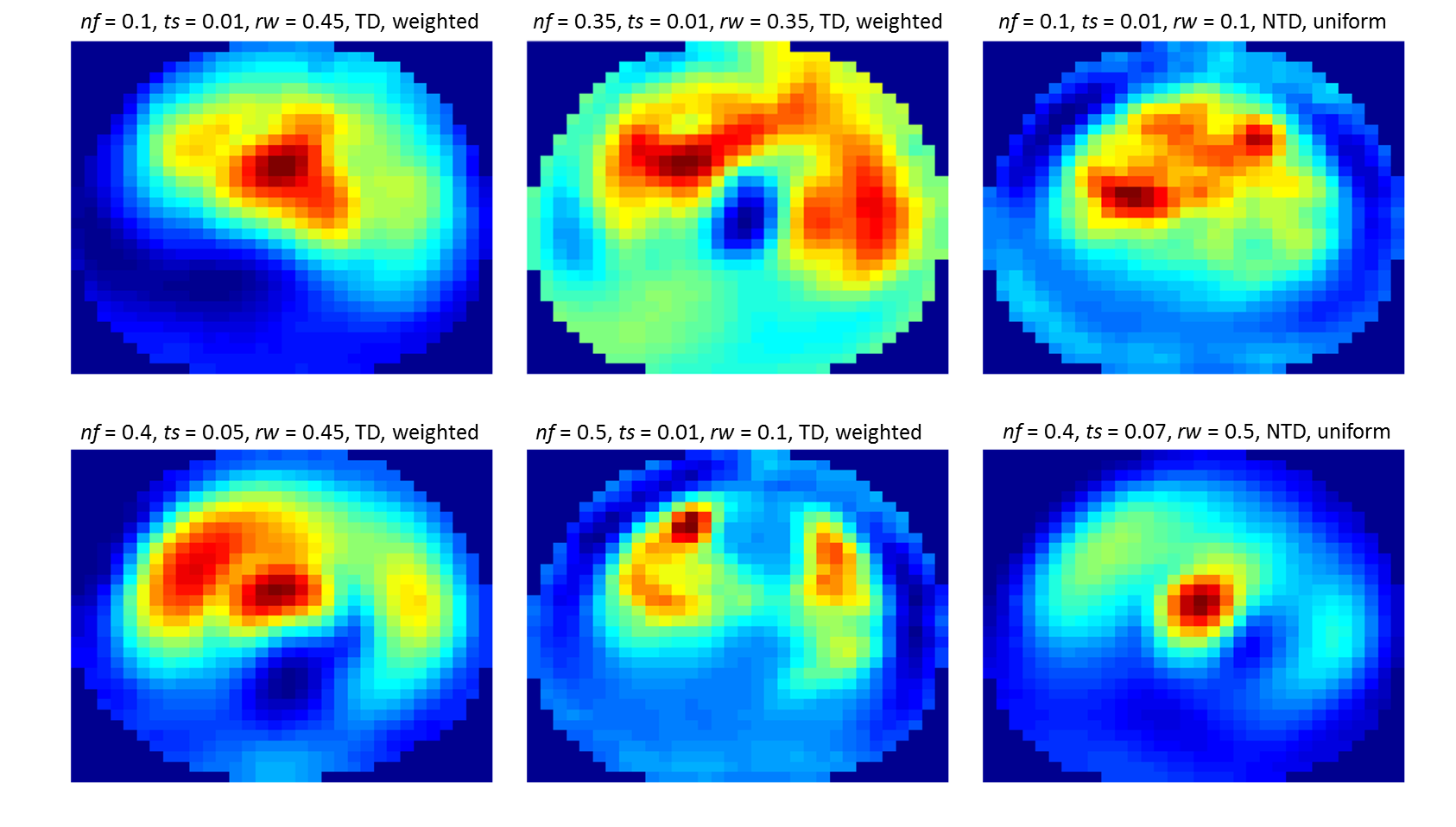

Supplement: S2 Fig — A collection of unphysiological EIT-images for certain reconstruction settings. Especially combinations containing rw below 0.1 and above 0.3 often generated distorted images from our data. (TIF) [file pone.0182215.s002.tif]

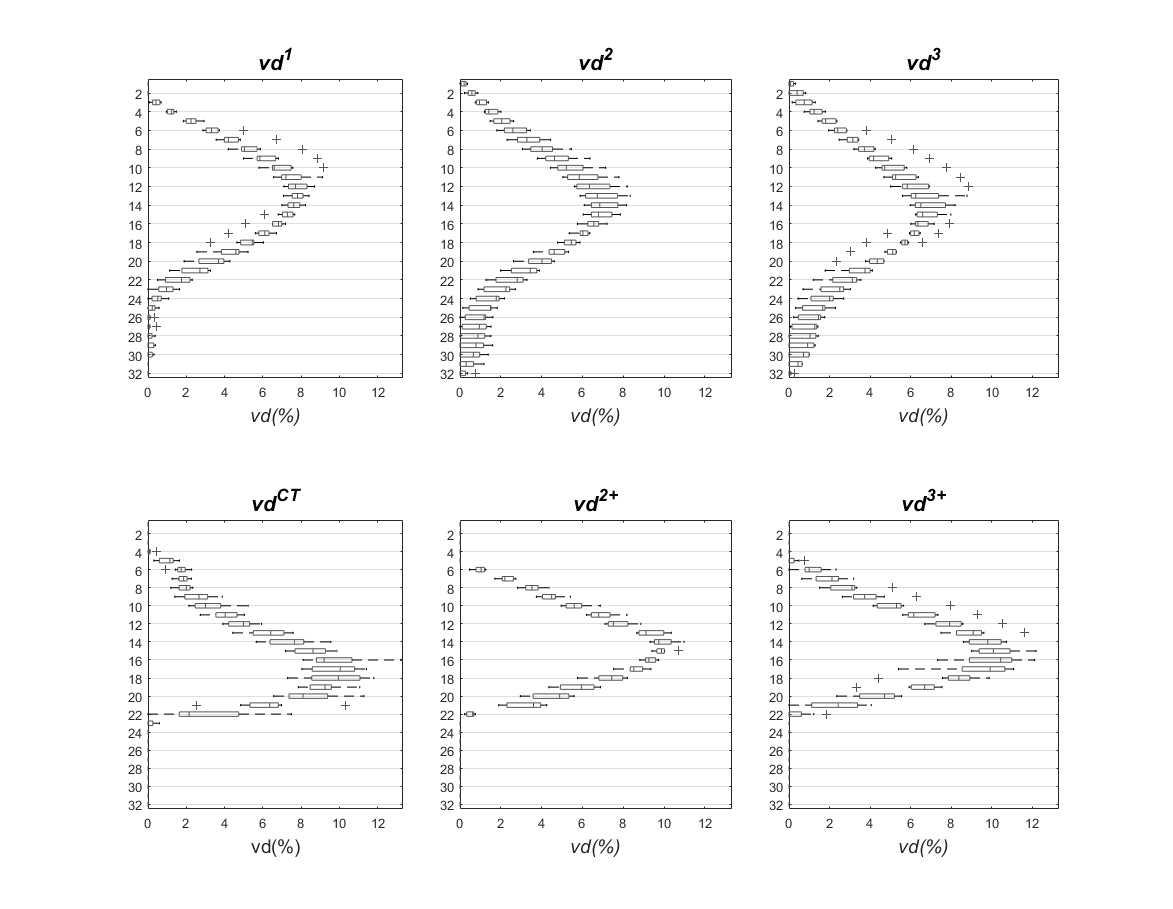

Supplement: S3 Fig — Data distribution of anteroposterior profiles for CT and EIT. Boxplots are given as median and 25th and 75th percentiles, respectively. (TIF) [file pone.0182215.s003.tif]

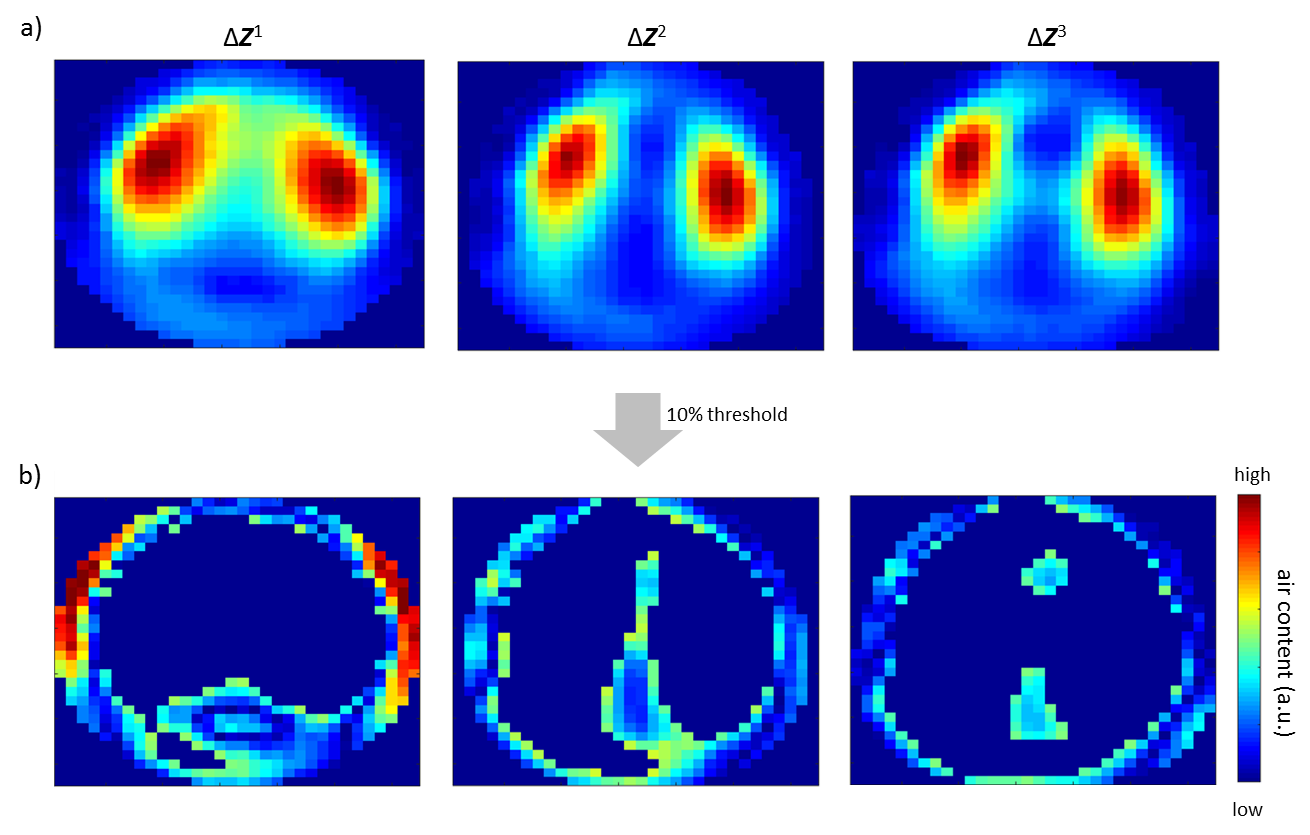

Supplement: S4 Fig — a) Tidal volume images (animal P06) for circular (ΔZ1), averaged (ΔZ2) and individualized (ΔZ3) reconstruction model. b) After truncating the image above 10% of the maximum value, noise levels at the boundary become visible. The noise images are rectified for better visualization of noise levels (the color bar is only valid for (b)). (TIF) [file pone.0182215.s004.tif]

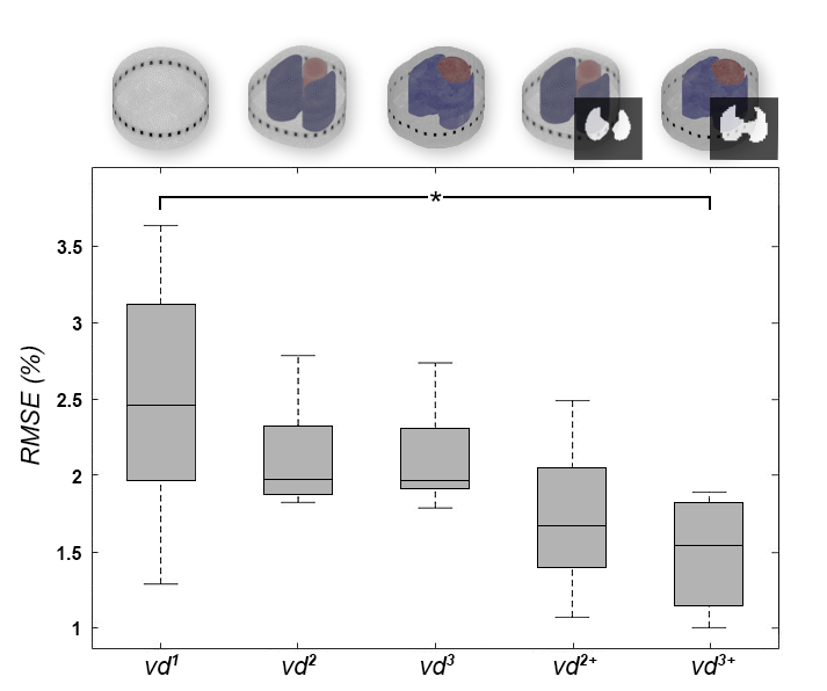

Supplement: S5 Fig — Boxplots for RMSE values over all pigs (n = 8) using GREIT with nf = 0.15, ts = 0.05, rw = 0.25, TD and weighted lungs and heart. The circular model (vd1) showed high variation and high error, whereas RMSE decreased with the addition of anatomical information in vd2, vd2+ and vd3. RMSE was significantly lower after adding further individual anatomical information in vd3+ (p < 0.04). (TIF) [file pone.0182215.s005.tif]
