# Supplementary material for: Effects of individualized electrical impedance tomography and image reconstruction settings upon the assessment of regional ventilation distribution: Comparison to 4-dimensional computed tomography in a porcine model
Source: PLoS One. 2017 Aug 1;12(8):e0182215. doi: 10.1371/journal.pone.0182215 (PMC5538699; doi:10.1371/journal.pone.0182215)
Supplement: S1 Table — Different reconstruction settings ranked by their 2d correlation values with 4DCT. Averaging the ranks of these settings provides a robust candidate for further analysis. (DOCX) [file pone.0182215.s006.docx]

| animal | algorithm | *nf* | *ts* | *rw* | ref | background | prior | correlation | rank |
| --- | --- | --- | --- | --- | --- | --- | --- | --- | --- |
| Pre01 | GN | 0.35 |  |  | TD | weighted | Tikonhov | 0.721 | 1 |
|  | GN | 0.4 |  |  | TD | weighted | Tikonhov | 0.720 | 2 |
|  | GREIT | 0.1 | 0.09 | 0.15 | TD | weighted |  | 0.705 | 3 |
|  | GREIT | 0.15 | 0.09 | 0.15 | TD | weighted |  | 0.705 | 4 |
|  | GREIT | 0.15 | 0.08 | 0.15 | TD | weighted |  | 0.702 | 5 |
|  | GREIT | 0.1 | 0.08 | 0.15 | TD | weighted |  | 0.702 | 6 |
|  | GN | 0.45 |  |  | TD | weighted | Tikonhov | 0.700 | 7 |
|  | GREIT | 0.1 | 0.07 | 0.15 | TD | weighted |  | 0.696 | 8 |
|  | GREIT | 0.15 | 0.07 | 0.15 | TD | weighted |  | 0.694 | 9 |
|  | GN | 0.3 |  |  | TD | weighted | Tikonhov | 0.694 | 10 |
|  | **GREIT** | **0.1** | **0.06** | **0.15** | **TD** | **weighted** |  | **0.691** | **11** |
|  | GN | 0.3 |  |  | NTD | weighted | Tikonhov | 0.690 | 12 |
|  | GREIT | 0.1 | 0.05 | 0.15 | TD | weighted |  | 0.683 | 13 |
|  | GREIT | 0.15 | 0.06 | 0.15 | TD | weighted |  | 0.682 | 14 |
|  | GREIT | 0.1 | 0.04 | 0.15 | TD | weighted |  | 0.676 | 15 |
| Pre02 | GREIT | 0.1 | 0.01 | 0.15 | NTD | weighted |  | 0.714 | 1 |
|  | GREIT | 0.1 | 0.03 | 0.15 | NTD | weighted |  | 0.695 | 2 |
|  | GREIT | 0.15 | 0.01 | 0.15 | TD | weighted |  | 0.690 | 3 |
|  | GREIT | 0.1 | 0.02 | 0.15 | NTD | weighted |  | 0.690 | 4 |
|  | GREIT | 0.1 | 0.04 | 0.15 | NTD | weighted |  | 0.689 | 5 |
|  | GREIT | 0.1 | 0.01 | 0.2 | NTD | weighted |  | 0.688 | 6 |
|  | GREIT | 0.15 | 0.09 | 0.15 | NTD | weighted |  | 0.687 | 7 |
|  | GREIT | 0.15 | 0.08 | 0.15 | NTD | weighted |  | 0.686 | 8 |
|  | GREIT | 0.1 | 0.05 | 0.15 | NTD | weighted |  | 0.685 | 9 |
|  | **GREIT** | **0.15** | **0.06** | **0.15** | **TD** | **weighted** |  | **0.683** | **10** |
|  | GREIT | 0.1 | 0.01 | 0.15 | TD | weighted |  | 0.682 | 11 |
|  | GREIT | 0.15 | 0.07 | 0.15 | TD | weighted |  | 0.682 | 12 |
|  | GREIT | 0.15 | 0.07 | 0.15 | NTD | weighted |  | 0.682 | 13 |
|  | GREIT | 0.15 | 0.05 | 0.15 | TD | weighted |  | 0.681 | 14 |
|  | GREIT | 0.15 | 0.08 | 0.15 | TD | weighted |  | 0.680 | 15 |
| Pre03 | GREIT | 0.5 | 0.09 | 0.35 | TD | uniform |  | 0.573 | 1 |
|  | GREIT | 0.5 | 0.08 | 0.35 | TD | uniform |  | 0.571 | 2 |
|  | GREIT | 0.45 | 0.09 | 0.35 | TD | uniform |  | 0.569 | 3 |
|  | GREIT | 0.45 | 0.08 | 0.35 | TD | uniform |  | 0.566 | 4 |
|  | GREIT | 0.5 | 0.07 | 0.35 | TD | uniform |  | 0.565 | 5 |
|  | GREIT | 0.4 | 0.09 | 0.35 | TD | uniform |  | 0.564 | 6 |
|  | GREIT | 0.1 | 0.07 | 0.15 | NTD | weighted |  | 0.563 | 7 |
|  | GREIT | 0.15 | 0.07 | 0.15 | NTD | weighted |  | 0.563 | 8 |
|  | GREIT | 0.45 | 0.07 | 0.35 | TD | uniform |  | 0.562 | 9 |
|  | GREIT | 0.4 | 0.08 | 0.35 | TD | uniform |  | 0.560 | 10 |
|  | GN | 0.25 |  |  | NTD | weighted | Tikonhov | 0.560 | 11 |
|  | GREIT | 0.4 | 0.07 | 0.35 | TD | uniform |  | 0.556 | 12 |
|  | GREIT | 0.35 | 0.09 | 0.35 | TD | uniform |  | 0.554 | 13 |
|  | GN | 0.3 |  |  | NTD | weighted | Tikonhov | 0.553 | 14 |
|  | **GREIT** | **0.15** | **0.06** | **0.15** | **TD** | **weighted** |  | **0.553** | **15** |
| Average  Rank | **GREIT** | **0.15** | **0.06** | **0.15** | **TD** | **weighted** |  | **0.639** | **12** |
|  | GREIT | 0.15 | 0.07 | 0.15 | TD | weighted |  | 0.639 | 22.667 |
|  | GREIT | 0.1 | 0.01 | 0.15 | TD | weighted |  | 0.625 | 22.667 |
|  | GREIT | 0.15 | 0.07 | 0.15 | NTD | weighted |  | 0.619 | 25.667 |
|  | GREIT | 0.1 | 0.04 | 0.15 | TD | weighted |  | 0.627 | 27 |
|  | GREIT | 0.1 | 0.05 | 0.15 | TD | weighted |  | 0.628 | 28.333 |
|  | GREIT | 0.1 | 0.03 | 0.15 | TD | weighted |  | 0.625 | 29 |
|  | GREIT | 0.1 | 0.04 | 0.15 | NTD | weighted |  | 0.616 | 29.333 |
|  | GREIT | 0.1 | 0.02 | 0.15 | NTD | weighted |  | 0.619 | 29.667 |
|  | GREIT | 0.1 | 0.03 | 0.15 | NTD | weighted |  | 0.619 | 31 |
