# Supplementary material for: Effects of individualized electrical impedance tomography and image reconstruction settings upon the assessment of regional ventilation distribution: Comparison to 4-dimensional computed tomography in a porcine model
Source: PLoS One. 2017 Aug 1;12(8):e0182215. doi: 10.1371/journal.pone.0182215 (PMC5538699; doi:10.1371/journal.pone.0182215)
Supplement: S2 Table — Based on 4 animals of the model comparison group, a validation of the “optimal” reconstruction settings was performed in accordance with the previous evaluation. The previously identified settings appear within the top 10. (DOCX) [file pone.0182215.s007.docx]

| animal | algorithm | *nf* | *ts* | *rw* | ref | background | prior | correlation | rank |
| --- | --- | --- | --- | --- | --- | --- | --- | --- | --- |
| P01 | GREIT | 0.15 | 0.07 | 0.15 | NTD | weighted |  | 0.671 | 1 |
|  | GREIT | 0.1 | 0.07 | 0.15 | NTD | weighted |  | 0.670 | 2 |
|  | GREIT | 0.15 | 0.09 | 0.15 | NTD | weighted |  | 0.668 | 3 |
|  | GREIT | 0.15 | 0.08 | 0.15 | NTD | weighted |  | 0.668 | 4 |
|  | GREIT | 0.1 | 0.06 | 0.15 | NTD | weighted |  | 0.666 | 5 |
|  | GREIT | 0.1 | 0.01 | 0.15 | NTD | weighted |  | 0.664 | 6 |
|  | GREIT | 0.1 | 0.05 | 0.15 | NTD | weighted |  | 0.662 | 7 |
|  | GREIT | 0.1 | 0.01 | 0.2 | NTD | weighted |  | 0.658 | 8 |
|  | GREIT | 0.1 | 0.03 | 0.15 | NTD | weighted |  | 0.657 | 9 |
|  | GREIT | 0.1 | 0.04 | 0.15 | NTD | weighted |  | 0.656 | 10 |
|  | GREIT | 0.1 | 0.08 | 0.15 | NTD | weighted |  | 0.652 | 11 |
|  | GREIT | 0.1 | 0.06 | 0.2 | NTD | weighted |  | 0.651 | 12 |
|  | GREIT | 0.1 | 0.02 | 0.15 | NTD | weighted |  | 0.649 | 13 |
|  | GREIT | 0.1 | 0.07 | 0.2 | NTD | weighted |  | 0.647 | 14 |
|  | GREIT | 0.1 | 0.03 | 0.2 | NTD | weighted |  | 0.645 | 15 |
| P02 | **GREIT** | **0.15** | **0.06** | **0.15** | **TD** | **weighted** |  | **0.760** | **1** |
|  | GREIT | 0.15 | 0.05 | 0.15 | TD | weighted |  | 0.759 | 2 |
|  | GREIT | 0.15 | 0.09 | 0.2 | TD | weighted |  | 0.758 | 3 |
|  | GREIT | 0.1 | 0.04 | 0.15 | NTD | weighted |  | 0.757 | 4 |
|  | GREIT | 0.15 | 0.08 | 0.2 | TD | weighted |  | 0.756 | 5 |
|  | GREIT | 0.15 | 0.04 | 0.15 | TD | weighted |  | 0.755 | 6 |
|  | GREIT | 0.1 | 0.05 | 0.15 | NTD | weighted |  | 0.755 | 7 |
|  | GREIT | 0.15 | 0.08 | 0.15 | NTD | weighted |  | 0.755 | 8 |
|  | GREIT | 0.15 | 0.07 | 0.15 | NTD | weighted |  | 0.754 | 9 |
|  | GREIT | 0.1 | 0.03 | 0.15 | NTD | weighted |  | 0.754 | 10 |
|  | GREIT | 0.15 | 0.07 | 0.2 | TD | weighted |  | 0.754 | 11 |
|  | GREIT | 0.15 | 0.03 | 0.15 | TD | weighted |  | 0.753 | 12 |
|  | GREIT | 0.15 | 0.09 | 0.15 | NTD | weighted |  | 0.752 | 13 |
|  | GREIT | 0.1 | 0.06 | 0.15 | NTD | weighted |  | 0.752 | 14 |
|  | GREIT | 0.1 | 0.01 | 0.15 | NTD | weighted |  | 0.751 | 15 |
| P03 | GREIT | 0.15 | 0.01 | 0.15 | TD | weighted |  | 0.658 | 1 |
|  | GREIT | 0.15 | 0.02 | 0.15 | TD | weighted |  | 0.651 | 2 |
|  | GREIT | 0.15 | 0.03 | 0.15 | TD | weighted |  | 0.646 | 3 |
|  | GREIT | 0.15 | 0.04 | 0.15 | TD | weighted |  | 0.643 | 4 |
|  | GREIT | 0.15 | 0.05 | 0.15 | TD | weighted |  | 0.642 | 5 |
|  | **GREIT** | **0.15** | **0.06** | **0.15** | **TD** | **weighted** |  | **0.641** | **6** |
|  | GREIT | 0.2 | 0.08 | 0.15 | TD | weighted |  | 0.636 | 7 |
|  | GREIT | 0.2 | 0.09 | 0.15 | TD | weighted |  | 0.636 | 8 |
|  | GREIT | 0.15 | 0.07 | 0.15 | TD | weighted |  | 0.636 | 9 |
|  | GREIT | 0.1 | 0.05 | 0.15 | NTD | weighted |  | 0.636 | 10 |
|  | GREIT | 0.15 | 0.08 | 0.15 | NTD | weighted |  | 0.635 | 11 |
|  | GREIT | 0.1 | 0.04 | 0.15 | NTD | weighted |  | 0.634 | 12 |
|  | GREIT | 0.15 | 0.09 | 0.15 | NTD | weighted |  | 0.634 | 13 |
|  | GREIT | 0.1 | 0.06 | 0.15 | NTD | weighted |  | 0.634 | 14 |
|  | GREIT | 0.2 | 0.01 | 0.15 | TD | weighted |  | 0.634 | 15 |
| P04 | GREIT | 0.1 | 0.01 | 0.15 | TD | weighted |  | 0.687 | 1 |
|  | GREIT | 0.1 | 0.07 | 0.15 | NTD | weighted |  | 0.681 | 2 |
|  | GREIT | 0.15 | 0.07 | 0.15 | NTD | weighted |  | 0.681 | 3 |
|  | GREIT | 0.1 | 0.01 | 0.2 | TD | weighted |  | 0.678 | 4 |
|  | GREIT | 0.15 | 0.09 | 0.15 | NTD | weighted |  | 0.677 | 5 |
|  | GREIT | 0.1 | 0.01 | 0.15 | NTD | weighted |  | 0.677 | 6 |
|  | GREIT | 0.1 | 0.06 | 0.2 | NTD | weighted |  | 0.671 | 7 |
|  | GREIT | 0.1 | 0.08 | 0.15 | NTD | weighted |  | 0.668 | 8 |
|  | GREIT | 0.15 | 0.08 | 0.15 | NTD | weighted |  | 0.668 | 9 |
|  | GREIT | 0.1 | 0.09 | 0.15 | NTD | weighted |  | 0.668 | 10 |
|  | GREIT | 0.15 | 0.06 | 0.15 | NTD | weighted |  | 0.668 | 11 |
|  | GREIT | 0.1 | 0.01 | 0.25 | TD | weighted |  | 0.665 | 12 |
|  | GREIT | 0.1 | 0.01 | 0.2 | NTD | weighted |  | 0.662 | 13 |
|  | **GREIT** | **0.15** | **0.06** | **0.15** | **TD** | **weighted** |  | **0.661** | **14** |
|  | GREIT | 0.1 | 0.07 | 0.2 | NTD | weighted |  | 0.661 | 15 |
| Average  Rank | GREIT | 0.15 | 0.08 | 0.15 | NTD | weighted |  | 0.681 | 7 |
|  | GREIT | 0.15 | 0.09 | 0.15 | NTD | weighted |  | 0.683 | 7.5 |
|  | GREIT | 0.15 | 0.07 | 0.15 | NTD | weighted |  | 0.684 | 8.25 |
|  | GREIT | 0.1 | 0.05 | 0.15 | NTD | weighted |  | 0.676 | 13.25 |
|  | GREIT | 0.1 | 0.04 | 0.15 | NTD | weighted |  | 0.674 | 13.5 |
|  | GREIT | 0.1 | 0.06 | 0.15 | NTD | weighted |  | 0.676 | 14 |
|  | GREIT | 0.1 | 0.07 | 0.15 | NTD | weighted |  | 0.679 | 14.75 |
|  | GREIT | 0.1 | 0.01 | 0.15 | NTD | weighted |  | 0.678 | 15 |
|  | GREIT | 0.1 | 0.03 | 0.15 | NTD | weighted |  | 0.674 | 15.25 |
|  | **GREIT** | **0.15** | **0.06** | **0.15** | **TD** | **weighted** |  | **0.660** | **19** |
