# Supplementary material for: Effects of individualized electrical impedance tomography and image reconstruction settings upon the assessment of regional ventilation distribution: Comparison to 4-dimensional computed tomography in a porcine model
Source: PLoS One. 2017 Aug 1;12(8):e0182215. doi: 10.1371/journal.pone.0182215 (PMC5538699; doi:10.1371/journal.pone.0182215)
Supplement: S3 Table — Individual error of model geometries for circular M1, mean M2 and individual M3 models. The error is defined as symmetric difference ΔS; i.e. non-overlapping regions of thorax contours divided by total area. As expected, ΔS was higher between M1 and M3 than between M2 and M3. All values are given in %. (DOCX) [file pone.0182215.s008.docx]

|  | M^1^ | M^2^ | M^3^ | | | | | | | |
| --- | --- | --- | --- | --- | --- | --- | --- | --- | --- | --- |
|  | **Circular** | **Mean** | **P01** | **P02** | **P03** | **P04** | **P05** | **P06** | **P07** | **P08** |
| **Circular** | 0.00 | 8.61 | 6.11 | 7.17 | 8.47 | 4.92 | 9.39 | 10.11 | 7.56 | 7.56 |
| **Mean** |  | 0.00 | 4.08 | 5.10 | 4.33 | 4.91 | 5.82 | 4.61 | 4.08 | 7.09 |
| **P01** |  |  | 0.00 | 3.52 | 5.68 | 3.80 | 7.55 | 6.91 | 2.55 | 6.40 |
| **P02** |  |  |  | 0.00 | 4.59 | 4.89 | 7.42 | 5.05 | 4.12 | 3.90 |
| **P03** |  |  |  |  | 0.00 | 6.73 | 6.15 | 3.04 | 6.39 | 4.57 |
| **P04** |  |  |  |  |  | 0.00 | 6.50 | 8.08 | 3.74 | 6.32 |
| **P05** |  |  |  |  |  |  | 0.00 | 5.77 | 7.69 | 5.98 |
| **P06** |  |  |  |  |  |  |  | 0.00 | 7.83 | 5.25 |
| **P07** |  |  |  |  |  |  |  |  | 0.00 | 6.89 |
| **P08** |  |  |  |  |  |  |  |  |  | 0.00 |
