# Supplementary material for: Effects of individualized electrical impedance tomography and image reconstruction settings upon the assessment of regional ventilation distribution: Comparison to 4-dimensional computed tomography in a porcine model
Source: PLoS One. 2017 Aug 1;12(8):e0182215. doi: 10.1371/journal.pone.0182215 (PMC5538699; doi:10.1371/journal.pone.0182215)
Supplement: S4 Table — Summary of average and standard deviation of anteroposterior ventilation distribution calculated from tidal volume images acquired by 4DCT and different EIT Models. All values are given in % as the fraction of horizontal region of interest (roi) ventilation to total ventilation. Approximate center of ventilations are written in bold letters. (DOCX) [file pone.0182215.s009.docx]

|  | *vd*^CT^ | | *vd*^1^ | | *vd*^2^ | | *vd*^3^ | | *vd*^2+^ | | *vd*^3+^ | |
| --- | --- | --- | --- | --- | --- | --- | --- | --- | --- | --- | --- | --- |
| roi | **mean** | **std** | **mean** | **std** | **mean** | **std** | **mean** | **std** | **mean** | **std** | **mean** | **std** |
| 1 | 0.0 | 0.0 | 0.0 | 0.0 | 0.2 | 0.1 | 0.1 | 0.1 | 0.0 | 0.0 | 0.0 | 0.0 |
| 2 | 0.0 | 0.0 | 0.0 | 0.0 | 0.6 | 0.2 | 0.4 | 0.3 | 0.0 | 0.0 | 0.0 | 0.0 |
| 3 | 0.0 | 0.0 | 0.4 | 0.2 | 1.0 | 0.3 | 0.7 | 0.4 | 0.0 | 0.0 | 0.0 | 0.0 |
| 4 | 0.1 | 0.1 | 1.2 | 0.2 | 1.5 | 0.3 | 1.3 | 0.4 | 0.0 | 0.0 | 0.0 | 0.0 |
| 5 | 1.0 | 0.5 | 2.3 | 0.4 | 2.1 | 0.5 | 1.9 | 0.4 | 0.0 | 0.0 | 0.2 | 0.3 |
| 6 | 1.7 | 0.4 | 3.5 | 0.7 | 2.7 | 0.6 | 2.6 | 0.6 | 1.0 | 0.3 | 1.1 | 0.7 |
| 7 | 1.8 | 0.3 | 4.5 | 1.0 | 3.3 | 0.7 | 3.3 | 0.8 | 2.3 | 0.4 | 2.0 | 0.8 |
| 8 | 1.9 | 0.4 | 5.4 | 1.2 | 4.1 | 0.8 | 4.0 | 0.9 | 3.5 | 0.5 | 2.9 | 1.1 |
| 9 | 2.6 | 0.8 | 6.3 | 1.2 | 4.8 | 0.8 | 4.6 | 1.0 | 4.4 | 0.5 | 3.9 | 1.1 |
| 10 | 3.2 | 1.0 | 7.0 | 1.0 | 5.5 | 0.9 | 5.3 | 1.1 | 5.7 | 0.6 | 5.3 | 1.2 |
| 11 | 4.0 | 0.8 | 7.5 | 0.8 | 6.1 | 1.0 | 5.8 | 1.2 | 6.9 | 0.7 | 6.7 | 1.2 |
| 12 | 4.9 | 0.7 | **7.8** | 0.6 | 6.6 | 1.0 | 6.3 | 1.2 | 7.8 | 0.6 | 8.1 | 1.2 |
| 13 | 6.3 | 1.1 | **7.8** | 0.4 | **6.9** | 0.9 | 6.7 | 1.1 | 9.3 | 0.7 | 9.1 | 1.2 |
| 14 | 7.4 | 1.3 | 7.6 | 0.4 | **7.0** | 0.8 | **6.9** | 0.9 | **9.9** | 0.6 | 9.7 | 0.8 |
| 15 | 8.5 | 1.0 | 7.2 | 0.5 | 6.9 | 0.6 | **6.8** | 0.6 | **9.9** | 0.4 | **10.2** | 1.1 |
| 16 | **9.8** | 1.6 | 6.6 | 0.7 | 6.5 | 0.4 | 6.6 | 0.6 | 9.3 | 0.3 | **10.0** | 1.5 |
| 17 | **9.8** | 1.3 | 5.9 | 0.8 | 6.0 | 0.3 | 6.2 | 0.7 | 8.6 | 0.5 | 9.3 | 1.9 |
| 18 | 9.8 | 1.6 | 5.1 | 0.9 | 5.4 | 0.4 | 5.6 | 0.8 | 7.3 | 0.8 | 8.1 | 1.6 |
| 19 | 9.2 | 1.0 | 4.3 | 0.8 | 4.6 | 0.6 | 4.9 | 0.8 | 5.8 | 0.9 | 6.3 | 1.3 |
| 20 | 8.4 | 1.5 | 3.3 | 0.9 | 3.9 | 0.7 | 4.1 | 0.8 | 4.5 | 1.0 | 4.3 | 1.2 |
| 21 | 6.2 | 2.2 | 2.5 | 0.8 | 3.2 | 0.7 | 3.4 | 0.8 | 3.2 | 0.9 | 2.2 | 1.4 |
| 22 | 3.1 | 2.4 | 1.6 | 0.7 | 2.5 | 0.8 | 2.8 | 0.8 | 0.5 | 0.2 | 0.4 | 0.7 |
| 23 | 0.1 | 0.2 | 0.9 | 0.5 | 1.9 | 0.7 | 2.2 | 0.8 | 0.0 | 0.0 | 0.0 | 0.0 |
| 24 | 0.0 | 0.0 | 0.5 | 0.4 | 1.5 | 0.6 | 1.7 | 0.8 | 0.0 | 0.0 | 0.0 | 0.0 |
| 25 | 0.0 | 0.0 | 0.2 | 0.2 | 1.1 | 0.7 | 1.4 | 0.7 | 0.0 | 0.0 | 0.0 | 0.0 |
| 26 | 0.0 | 0.0 | 0.1 | 0.1 | 0.9 | 0.6 | 1.1 | 0.6 | 0.0 | 0.0 | 0.0 | 0.0 |
| 27 | 0.0 | 0.0 | 0.1 | 0.1 | 0.8 | 0.6 | 0.9 | 0.6 | 0.0 | 0.0 | 0.0 | 0.0 |
| 28 | 0.0 | 0.0 | 0.1 | 0.1 | 0.7 | 0.6 | 0.8 | 0.7 | 0.0 | 0.0 | 0.0 | 0.0 |
| 29 | 0.0 | 0.0 | 0.1 | 0.2 | 0.7 | 0.6 | 0.7 | 0.6 | 0.0 | 0.0 | 0.0 | 0.0 |
| 30 | 0.0 | 0.0 | 0.1 | 0.1 | 0.6 | 0.5 | 0.6 | 0.5 | 0.0 | 0.0 | 0.0 | 0.0 |
| 31 | 0.0 | 0.0 | 0.0 | 0.0 | 0.4 | 0.4 | 0.4 | 0.3 | 0.0 | 0.0 | 0.0 | 0.0 |
| 32 | 0.0 | 0.0 | 0.0 | 0.0 | 0.2 | 0.3 | 0.1 | 0.1 | 0.0 | 0.0 | 0.0 | 0.0 |
